# Integrated phenotypes: understanding trait covariation in plants and animals

**DOI:** 10.1098/rstb.2013.0245

**Published:** 2014-08-19

**Authors:** W. Scott Armbruster, Christophe Pélabon, Geir H. Bolstad, Thomas F. Hansen

**Affiliations:** 1 School of Biological Sciences, University of Portsmouth, Portsmouth PO12DY, UK; 2 Institute of Arctic Biology, University of Alaska, Fairbanks, AK 99775, USA; 3 Department of Biology, Norwegian University of Science and Technology, 7491 Trondheim, Norway; 4 Center for Biodiversity Dynamics, Department of Biology, Norwegian University of Science and Technology, 7491 Trondheim, Norway; 5 Centre for Ecological and Evolutionary Synthesis, Department of Biology, University of Oslo, PO Box 1066, 0316 Oslo, Norway

**Keywords:** integration, modularity, variation, phenotype

## Abstract

Integration and modularity refer to the patterns and processes of trait interaction and independence. Both terms have complex histories with respect to both conceptualization and quantification, resulting in a plethora of integration indices in use. We review briefly the divergent definitions, uses and measures of integration and modularity and make conceptual links to allometry. We also discuss how integration and modularity might evolve. Although integration is generally thought to be generated and maintained by correlational selection, theoretical considerations suggest the relationship is not straightforward. We caution here against uncontrolled comparisons of indices across studies. In the absence of controls for trait number, dimensionality, homology, development and function, it is difficult, or even impossible, to compare integration indices across organisms or traits. We suggest that care be invested in relating measurement to underlying theory or hypotheses, and that summative, theory-free descriptors of integration generally be avoided. The papers that follow in this Theme Issue illustrate the diversity of approaches to studying integration and modularity, highlighting strengths and pitfalls that await researchers investigating integration in plants and animals.

## 1. Introduction

Variation is a fundamental property of life. However, phenotypic traits do not vary independently, but instead reflect webs of developmental, physiological and functional interactions of varying strengths [1–3]. Interest in the covariation of phenotypic traits has a long history in evolutionary biology, dating back at least as far as Darwin's discussion of multi-trait correlations in domestic animals ([4], ch. 1). The concept of morphological integration came into common use following Olson & Miller's seminal work [5] on patterns of covariation among traits and their relationships to functional needs. Olson and Miller did not formally define the term integration and used it to refer to both statistical correlations and functional interactions [6,7]. The terms phenotypic and morphological integration have thus acquired very broad usage, covering both observed patterns of covariation, the capacity or tendency for covariation, the underlying organismal architecture that gives rise to the tendencies and, ultimately, to the observed patterns and their evolutionary causes and consequences.

We may thus recognize several distinct conceptual variants of integration. These include (i) statistical or phenomenological integration, understood as patterns of strong phenotypic or genetic correlations in standing population variation; (ii) variational integration, defined as a tendency for covariation, as proposed by Hallgrímsson *et al*. [8]; (iii) developmental or structural integration of organismal architecture, which includes developmental interactions and phenomena such as the partial or complete fusion of parts. We may also recognize (iv) functional integration, which refers to parts of the organism that function together as a unit and (v) evolutionary integration, which refers to sets of traits with a disposition for evolving as a unit. See also Klingenberg [9] for a similar classification.

**Figure 1.**
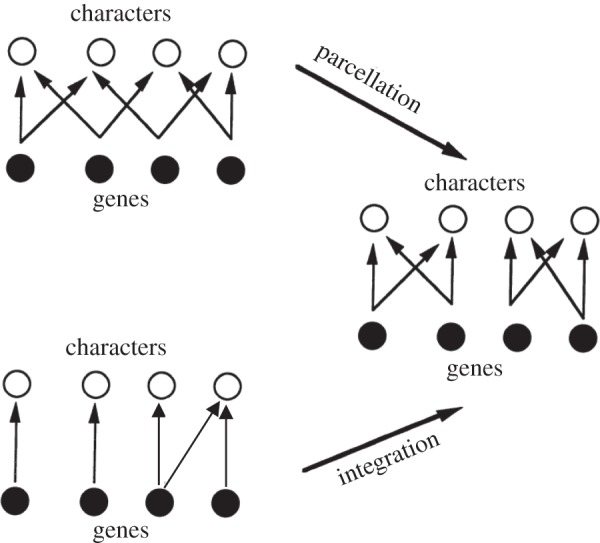
Potential evolutionary routes to modularity via parcellation and via increased integration (modified from Wagner & Altenberg [13]). In the case of parcellation, pleiotropic links between modules are removed, whereas with increased integration pleiotropic links are added within the modules, so as to make them relatively more integrated than the whole.

The concept of integration is closely related to the concept of modularity, which likewise lacks a single definition and is used in a variety of contexts. Like integration, modularity can refer to patterns of standing (co)variation, variational independence, developmental/structural independence or evolutionary independence among sets of traits [10,11]. Raff [12] defined (developmental) modularity in terms of the contextual independence of a developmental process, as when a limb bud develops according to its own rules even when it is grafted to a different position or body. Wagner & Altenberg [13] defined variational modules as (clusters of) phenotypic traits that have a disposition for internal covariation (integration), but are relatively independent of other such clusters (figure 1). Hence, modules are (integrated) processes or traits with a relative lack of integration with the rest of the organism.

Integration has particularly commanded the interest of modern evolutionary biologists because of its potential to constrain the course of phenotypic evolution. Indeed, Lewontin [14] postulated that a degree of ‘quasi-independence’ (i.e. evolutionary modularity) of characters was a necessary prerequisite for adaptive evolution to happen. The idea of integration as a constraint is reflected in such concepts as evolutionary lines of least resistance [15], in the idea of allometric constraints or co-regulation of trait growth as constraints [16–20], in the idea of evolution as correlated progression [21], and in the concept of conditional evolvability [22], the last of which may be regarded as a quantification of quasi-independence.

While increased modularity is generally seen as enhancing the evolvability of the module in question (but see [23,24]), integration is not necessarily just a negative constraint. In particular, Gould [16,25] has argued that constraints may often play a positive role by channelling variation in directions where selective challenges are likely to arise, or by creating ‘spandrels’ that can serve as substrate for new adaptations [25]. Hence, integration may also be a facilitator of adaptation (see also Riedl [26]). This is particularly easy to imagine when parts of the organism need to function together in a coordinated manner. It then benefits the organism if these parts are variationally linked, because this will reduce maladaptive uncoordinated variation, and it may also benefit the population by facilitating adaptive changes.

A key question is thus whether integration (or modularity) can evolve as some form of adaptation. The most obvious candidates here are as population-level adaptations for evolvability, by facilitating coordinated variation for selection to act upon, or as adaptation for robustness, by reducing potentially maladaptive genetic or environmental variation. Alternatively, integration may evolve in a non-adaptive manner as side-effect of selection on traits or on the efficiency and accuracy of the developmental system. Many of the pioneers in the study of integration and modularity were interested in them as potential adaptations [5,26–36]. However, it is not a trivial task to assess what mode of selection may cause a given pattern of integration. We explore these issues in §3.

Integration is linked conceptually to the extensive literature on allometry ([37,38]; see [9,18,19]), in which similarly opposing views on constraint versus adaptation have also arisen [20]. Allometry is a special case of developmental integration, because similar explicit underlying developmental or functional processes are used to explain the proportional size variation between two or more traits [37,39]. While some authors have considered allometry as a possible constraint for evolution resulting from the developmental process [16,17,38], other have argued that allometry is itself evolvable, the trajectory being maintained by selection [40–43].

## 2. Definitions and use of ‘integration’ and ‘modularity’

One of the most confusing issues surrounding the study of biological integration is the lack of consensus about a proper theoretical framework. The result is a diversity of different uses, definitions and methods of measurement.

### (a) ‘Statistical’ integration

The concept of integration is often used synonymously with correlation or pattern of correlation. One may view this as simply repackaging a study of correlations, but it may also reflect an expectation of direct links between observed patterns of variation and underlying developmental and physiological structure. However, this may also be naive. One of the fundamental discoveries of evolutionary quantitative genetics in the 1990s was that underlying structure cannot be inferred from patterns of variation. For example, it was realized that fundamental trade-offs between traits do not necessarily, or even usually, lead to negative phenotypic or genetic correlations, because they can be masked by variation in acquisition [44–47]. Hence, in lacking an explicit theoretical link to underlying organismal structure or to evolutionary consequences, the phenomenological concept of integration has little content beyond correlation. The term integration becomes mere window dressing for descriptive correlational studies, and we suggest that use in this context be avoided in the future.

### (b) Variational integration

Hallgrímsson *et al*. [8] suggested that integration should be treated as a dispositional concept in the same way as Wagner & Altenberg [13] suggested for the terms ‘variability’ and ‘evolvability’. Hence, just as variability refers to a tendency or disposition for variation and not to the expressed variation, integration can be defined as a tendency or disposition for covariation. This avoids the theoretical shallowness of using integration as another name for correlation by giving the concept a defined theoretical role. Examples of variational use of integration may be found in studies of patterns of pleiotropy (*genetic integration* in the terminology of Klingenberg [9]). Here, the focus is not on the expressed variation, which is also influenced by population history, patterns of selection, etc., but instead on the underlying potential. Studies of patterns of statistical ‘integration’ among new mutations often fall into this category. Following Wagner & Altenberg [13], the concept of modularity can also be used in a dispositional manner, referring to a potential for independent variation.

### (c) Developmental integration

Developmental integration refers to the underlying developmental and physiological mechanisms that create the disposition for covariation. These are mechanisms that can be studied experimentally [35,48]. Studies of developmental integration may thus be classified as studies of the genotype–phenotype map, with an emphasis on the causes of trait covariation. In this category, we find studies of hormonal systems and their ability to create coordinated variation in different traits [49–51], and studies of growth regulation and the developmental basis of allometric ‘integration’ [37,52–55]*.* There is also a number of formal models of how underlying developmental architecture is converted into trait (co)variation [46,56–60].

### (d) Functional integration

Functional integration is an important but challenging concept, because it encompasses proximal causes of phenotypic integration, ultimate causes of genetic integration and even the absence of detectable covariation within populations. Starting with Tedin [27]*,* Terentjev [28], Stebbins [29] and Olson & Miller [5], numerous authors have written about traits working together to perform some function [33,34,61–63]. Indeed, this is the underlying logic of most studies of phenotypic integration and one reason why integration is of interest to ecologists as well as developmental geneticists.

### (e) Evolutionary integration

The terms integration and modularity are also often used to refer to evolutionary dispositions. Hence, evolutionary integration is the disposition for two or more traits to evolve jointly during the divergence of populations or species. Evolutionary integration is thus related to the concept of evolvability. In the case of evolutionary modularity, this is expressed in Lewontin's [14] concept of quasi-independence, which was clearly intended as a dispositional concept. As discussed in §3, however, the relationship of integration and modularity (in any other sense) to evolvability is not simple and one-to-one.

## 3. Evolution of integration

Here, we first review the theoretical basis for the evolution of integration and modularity. We then provide a few empirical examples of evolutionary responses to apparent selection for integration in plants and animals.

### (a) Theoretical considerations

In discussing the evolution of integration, it is important to make clear what level of integration we are talking about [64,65]. Some of the literature on integration simply discusses the evolution of standing genetic or environmental variation in the population. In particular, the evolution of genetic variation commands a large literature, and we do not review the evolution of ‘statistical’ integration here. Instead, we focus on the evolution of variational and developmental integration. Before going into that, however, it is useful to make a few remarks on the effects of selection on standing variation.

It is well known that selection on variances and covariances depends on nonlinearities in the fitness surface. Positive second derivatives of fitness with respect to traits will increase variance, negative second derivatives will decrease variance, and for selection to alter the covariances between two traits, it is necessary that the joint second derivative of fitness with respect to the two traits is non-zero (correlational selection) [66]. Provided there is no skew in the trait distribution, a linear (flat) surface will only lead to weak reduction in variance that is second order in the strength of selection [67]. More generally, we can say that convexity in the fitness surface along a certain direction will favour variation in this direction, whereas concavity will disfavour variation. The relationship of this to integration is not straightforward however. First, if integration is conceptualized as a correlation at the population level, then there is no direct link to correlational selection, because change in correlations will depend on changes in both the covariance and the variances of the traits. Correlations will increase if selection increases covariances more than variances. Thus, the simple expectation of correlational selection favouring correlation needs to be qualified. Second, selection is not evolution. Even if the direct change in variation as a consequence of selection is precisely described by a simple model [66], the evolutionary responses of second moments are complicated and depend on the details of the genetic architecture [67].

A further complication is that selection on statistical integration is not transferred to selection on underlying variational or developmental integration in any simple manner, because the link between expressed variation and underlying architecture is not one-to-one, as discussed above. Most discussion of variational integration and modularity is couched in terms of patterns of pleiotropy. Hence, a good starting point for understanding the evolution of variational integration would be to understand the evolution of pleiotropy. Unfortunately, there is not much formal theoretical work on this question (but see below). Wagner [36] and Wagner & Altenberg [13] present some of the first explicit, although verbal, theories by discussing what selection pressures could lead to modularity by eliminating pleiotropic links between functional modules so as to make developmental modularity align with functional modularity. Wagner [36] dismissed constant stabilizing selection, including correlational selection as above defined, as a potent force for the evolution of modularity. Instead, Wagner suggested that modularity evolves by a combination of stabilizing and fluctuating directional selection where separate (functional) modules repeatedly find themselves under directional selection, whereas other modules are under stabilizing selection. This hypothesis remains to be tested with formal models.

Pleiotropy means that a gene, or more precisely an allelic substitution, affects more than one trait. Hence, the evolution of pleiotropy is a special case of the evolution of gene effects, and we may draw some insights from theoretical work on the evolution of gene effects on one-dimensional traits. This theory mostly concerns the evolution of canalization and we can relate the evolution of integration to canalization of the gene effects that do not fit the pattern of integration. Several hypotheses about the evolution of integration can be drawn from this. First, it is clear that the evolution of canalization depends on epistasis ([68–71], but see below)*,* and from this, we may infer that epistasis must be a central element for a working theory of the evolution of integration. Second, epistatic models show that either canalization or decanalization is a possible outcome of directional selection, depending on the directionality of epistasis [71,72]. This indicates that the evolution of integration may also depend on systematic patterns of gene interaction, and not just on mode of selection. Third, Waddington's [73] classic hypothesis that stabilizing selection leads to canalization also suggests that stabilizing selection may be important for integration, but this needs qualifications. Although formal analysis has shown that stabilizing selection indeed induces canalizing selection for reduced gene effects [60,68,69,74–76], the consequent reduction of standing variation also reduces the opportunity for stabilizing selection. The result is that the strongest canalization tends to evolve under stabilizing selection of intermediate strength [68,74]*.* Hence, integration may also evolve more easily for trait combinations under intermediate strengths of stabilizing selection. Finally, Le Rouzic *et al*. [74] found also that most forms of stationary fluctuating selection tend to induce canalizing selection, which suggests that fluctuating selection may also play a role in the evolution of integration, although this role may not be straightforward.

Hence, there emerges a picture of the evolution of integration and modularity with a complex relationship to mode and strength of selection, and an essential dependency on patterns of epistasis. There is a need for more theoretical work on the evolution of pleiotropy to achieve a robust predictive theory. Some simulation-based studies exist [77,78], but their interpretation is not straightforward (see Hansen [79] for critical review). Guillaume & Otto [80] provide a recent investigation of the conditions under which pleiotropy can evolve to fit functional trade-offs (see also Rueffler *et al*. [81]). Cheverud and co-workers have hypothesized that the evolution of integration requires differential epistasis, meaning different (directional) patterns of epistasis on different traits [82–84].

Although important, epistasis may not be the only basis for the evolution of pleiotropy, which can also happen through systematic modifications of the mutational spectrum by allele substitutions at a single locus [23,79]*.* For example, the evolution of pleiotropy could happen through gene duplication and subsequent subfunctionalization or neofuctionalization [80,85,86]. Wagner & Altenberg [13] conceptualized the evolution of patterns of integration in terms of adding or removing pleiotropic links (figure 1), and this could happen, for example, through the appearance or loss of *cis*-regulatory modules (*sensu* Wray *et al*. [87]).

Perhaps the main hypothesis that motivates many studies of integration and modularity is that patterns of integration will evolve to match functional relationships. The theoretical considerations above show that this is not an obvious prediction, although there are some results pointing in this direction [80,81,88]*.* It is also often unclear what a match between integration and function would be an adaptation for. Perhaps the most interesting possibility is that functional integration is an adaptation to increase evolvability by making the population more capable of evolving in likely directions of selection, because functionally related parts show coordinated variability. It is not obvious how this can happen by individual selection because evolvability is a population-level phenomenon, but it could plausibly happen through group or species selection [89]. Arguably, integration could also raise individual fitness by making an individual's offspring more likely to be adapted to spatial and/or temporal variation in the environment (especially in organisms with high fecundity and undirected dispersal of offspring). Adaptation for robustness is another option. The canalization of functionally uncoordinated variability may be favoured by individual selection through improving the precision of phenotypic expression. Armbruster and co-workers [63,90,91] have argued that adaptation consists of two components: optimization of the expected (average) phenotype and improved precision of its expression. Integration could benefit the latter. A final important possibility for adaptive evolution of integration is that it is an adaptation to improve environmental robustness and not genetic robustness. The latter may then follow as side-effect of the former (the ‘congruence’ hypothesis [68,92,93]).

It is also possible that integration evolves in a largely non-adaptive manner. Lynch [94,95] has argued that many aspects of genomic architecture are consequences of genetic drift as slightly deleterious changes of, for example, gene duplications cannot be effectively selected against in finite populations. With a different non-adaptive hypothesis, Hansen [79] has argued that the major factor likely to affect the evolution of variational properties is indirect selection stemming from trait adaptation. The consequence of this is that integration will evolve in a largely idiosyncratic manner depending on the genetic details of its relation to trait change. For example, a mutation creating a new pleiotropic link may be favoured simply because the new trait it affects is currently under directional selection, even if this creates an ‘unfavourable’ covariance with other traits [23]*.* Many have also argued that integration can be a side-effect of evolution of developmental interactions [59,79,96]. None of these scenarios predicts a close match between developmental and functional integration.

Regardless of the mode of evolution of pleiotropy and developmental integration, we wish to emphasize that a relationship between, for example, function and developmental integration does not necessarily transfer to a relationship between function and variational, evolutionary or statistical integration. For example, the patterns of pleiotropy that maximize the average evolvability of given trait modules are not generally modular [23,24], and standing covariance may fail to reflect both underlying trade-offs and modules in the genetic architecture [46,64,97].

### (b) Some empirical examples

Since the pioneering work of Olson & Miller [5], a huge number of studies of integration has accumulated. The field particularly took off with the emergence of evolutionary developmental biology and its emphasis on the genotype–phenotype map, and with the rapid development of ever more sophisticated morphometric methods (reviewed in [9,48,64,98–100]). Several important model systems have emerged to yield insights into the developmental basis and evolutionary consequences of integration. These include the mouse mandible and cranium [35,65,101–107], the eyespots on butterfly wings [108–112]*,* limbs and pelvises of hominids and other mammals [113–118]*,* the primate cranium [33,34,48,119–123], the insect wing [124–129] and many others. We do not attempt to review this here, but restrict ourselves to some general comments illustrated with the integration of angiosperm flowers, the important model system introduced by Berg [30,31].

Over half of the papers in this theme issue and a large proportion of the literature on integration concern the integration and/or modularity of flowers. The origins of this interest can be traced back to a pair of papers by Berg [30,31], which have stimulated much of the research on integration and modularity in plants and other organisms (see review by Murren [7] and discussion by Conner & Lande [130]). Despite the fact that Berg's papers focused primarily on homeostasis and modularity (‘correlation pleiades’), most of the subsequent literature stimulated by Berg's work has addressed patterns of integration [130]. This paradox reflects the fact that there are two divergent interpretations of her thinking that probably stemmed from the various ideas developed by Berg regarding the effects of specialized pollination on flowers. We illustrate this with the following quotations:

— ‘In the ten [specialized] species of plants possessing tubular flowers … a high positive correlation was observed between the dimensions of the reproductive parts … ’ [30, p. 104].
— ‘All plants with specific insect pollinators … have correlation pleiades. All plants lacking specific pollinators, be they self-pollinated, anemophiles or entomophiles without specialized insect pollinators, lack correlation pleiades’ [31, p. 176].
— ‘The adaptations to localize pollen deposition involve: [in addition to modularity] … the development of tubular parts, reducing the arena where the critical events take place; the reduction in number (oligimerization) and the rigid fixation of the number of homologous parts’ [31, p. 177].

**Figure 2.**
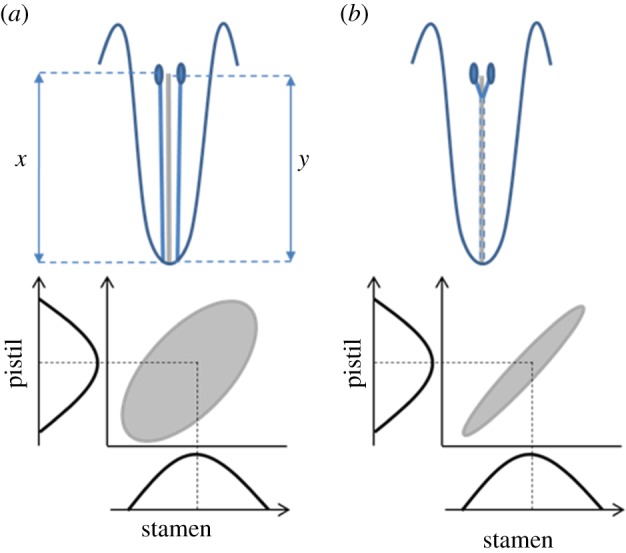
Increased floral integration, from unfused pistil and stamens (*a*), to adnate (structurally integrated) pistil and stamens (*b*). Fusion of filament and style tissues can lead to an increase or decrease in measured (statistical) phenotypic integration of pistil and stamen, depending, respectively, on whether the portions of the stamen filaments fused to the style (dotted lines) are, or are not, included in the stamen measurements. (The former analysis would depend on phylogenetic/evolutionary or developmental insights.) (Online version in colour.)

The first statement suggests that at least some flowers with specialized insect pollinators are highly integrated. The second statement has been interpreted to mean that flowers with specialized pollination are highly modularized, where floral modules could originate by integration and/or parcellation (figure 1). The first and third statements suggest that structural integration (e.g. corolla fusion and tubularity) is greater in flowers with specialized pollination than in flowers with less specialized pollination (see discussions by Armbruster *et al*. [131] and Conner & Lande [130]). Additionally, Berg's *a priori* classification of flowers with fused parts as specialized (see third statement) also lends support to the idea that she thought that specialized flowers should show greater structural integration. In Berg's view, increasing integration was achieved by the reduction of floral parts via fusion (third statement), and not by simple stabilizing selection on the different parts, as often suggested in the literature. Strong selection for covariation between two organs may lead to their fusion as a mechanism to maintain their functional and variational coherence and will promote their statistical integration in the face of genetic and environmental variation (figure 2).

Whether Berg imagined only parcellation or instead both parcellation and integration in the evolution of specialized modular flowers may never be resolved (but see discussion by Conner & Lande [130]). Nevertheless, both ideas have been tested and have received some support, but the debate is still ongoing as illustrated by the collection of papers in this theme issue.

This fusion, or developmental (structural) integration, may result in the near-perfect fit of individual and population means onto the governing adaptive ridge [132]. This was originally suggested by Stebbins [133,134], who argued that connation (fusion of parts from the same floral whorl) and adnation (fusion of parts from different floral whorls) represented a major trend in flowering plants, largely responsible for the type of ‘simplified’ (fewer parts, highly fused) flowers most often seen today (see also [135]). The effect of organ fusion on statistical integration is illustrated in figure 2, where we consider the covariance between two traits defining the position of the anther (pollen) and stigma in a flower relative to a common landmark (e.g. the nectary). In figure 2*a*, the covariance between the length of the pistil and filament results from the part of the variation they have in common during their development, which correspond to the general size of the flower, whereas trait-specific growth and developmental noise will decrease their correlation. Fusing pistil and filament into a common structure in order to increase the accuracy of pollination by limiting the variation in the position of the anther and stigma will transform a large amount of the variation in each trait (variation in trait size and developmental noise) into covariation (figure 2*b*). Whether or not such developmental integration can be detected statistically will depend on the definition of the traits.

**Figure 3.**
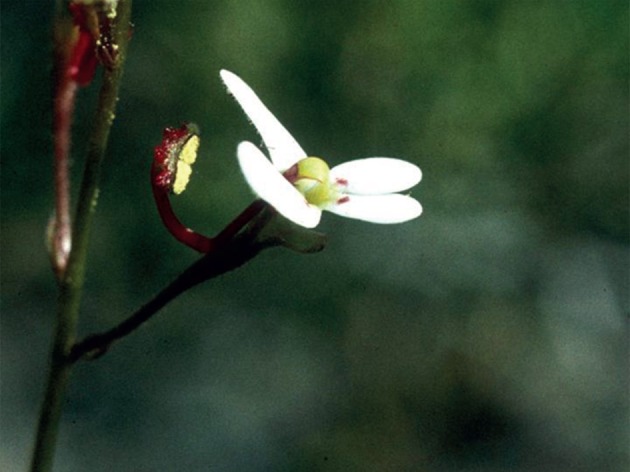
Flower of *Stylidium bicolor* in the staminate phase. The column bearing the pollen is formed by fusion (adnation) of staminate and pistillate tissues and will bear the stigma in place of the pollen in one or two days. Here, comparing column length in the male and female phases shows that the positions of the anthers and stigmas are tightly correlated because of the structural integration. (Online version in colour.)

The fusion of male and female parts into a single column bearing either the anthers or, later in floral development, the stigmas in *Stylidium* (Stylidiaceae) flowers illustrates perfectly this issue, because pistil and filament tissues cannot be distinguished from each other (figure 3). In this case, near-perfect covariance between stamen and style is achieved by fusion, but to detect this requires measuring the same structure twice, once for each function, which are temporally sequential [90,132,136]. But if the fusion is imperceptible and unrecognized, as may often be the case in flowers, for example, petal–filament fusion (epipetally), then the portion measured will be structurally and potentially statistically independent of the rest of the other structures.

Considering the increased integration expected among floral parts in species with specialized pollination, we have noted a conceptual divergence that has developed in the literature. Many studies have explored the expectation that all floral parts should be integrated and flowers modular (decoupled from vegetative traits) when they are adapted for specialized pollination. A second, perhaps more realistic, expectation is that only certain floral traits (e.g. pollination efficiency and pollinator fit traits, such as style and stamen lengths) are correlated, whereas others (e.g. floral advertisement traits, such as petal size) may not be [61,131,135,137,138]*.* Consequently, flowers may comprise several modules, with, for example, pollen-transfer traits and advertisement traits representing two partly independent units nested with the module represented by the flower as a whole [138,139]. Finally, not all floral traits necessarily belong to the floral module (for example, sepals or pistils may be correlated with vegetative traits [131]; cf. [140]).

In general, flowers, like the vertebrate skull (see [141,142]), appear to show integration between some organs, including by fusion, as well as lack of integration (and often modularity) between other organs. As Armbruster *et al*. [63,131] and Ordano *et al*. [137] have pointed out, integration of flowers is best studied in the light of functional or developmental hypotheses, and measures of overall statistical integration are not very informative unless possible modular organization is taken into account.

## 4. A multi-level perspective on integration

In considering integration at multiple levels of organization, it is important to recognize the distinction between levels and causes, where the former are units of organization and analysis and the latter are sources of variance and covariance (see Klingenberg [143]). It is these causes that generate the integration patterns at each level, but the causes are not necessarily in operation at all levels [62]. For example, Armbruster [144] distinguished between causal functions generating variation and covariation among blossoms within genetic individuals (genets), among genets, and among populations of one widespread plant species. Armbruster hypothesized that variance and covariance within genets were caused by variation in the local environment and in ontogenetic stage, whereas variation and covariation at the among-genet level were *additionally* influenced by genetic variation, and variation and covariation among populations were *additionally* affected by evolutionary divergence. These can be viewed as emergent processes at the level of organization at which they first appear. The emergent processes can be detected and estimated by partitioning out upper-level variances and covariances from the total variances and covariances by modifying models developed for nested analysis of variance (i.e. using ‘nested analysis of covariance’; [62,144–146]) or by using contextual analysis [147,148].

**Figure 4.**
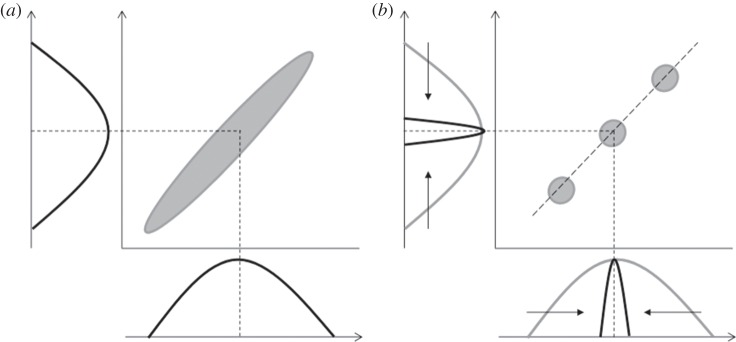
(*a*,*b*) Loss of detectable covariance with stabilizing selection acting together with correlational selection. Although populations will evolve only along the ridge (diagonal dashed line), the variance within each population is too small relative to the width of the adaptive ridge, for any within-population covariation to be detected.

Multi-level approaches are also important, because stabilizing selection may reduce the genetic variance (and covariance) in a population, and a single population may therefore provide few signs of integration, despite strong functional or developmental integration being in place. Statistical integration (trait covariation) is then revealed only when multiple populations are compared (figure 4). For example, in *Dalechampia* vines (Euphorbiaceae), an estimated pollination-related adaptive surface governing the shape and size of blossoms leads to the expectation that the amount of pollinator reward reflected in the resin-gland area covaries with the distance between the gland and the stigma. This is because the gland area influences the size of the largest bee visitors and gland–stigma distance influences the size of the smallest bee pollinators [145,149]. This relationship between trait functions almost certainly generates correlational selection [62,63,135], but neither phenotypic covariation nor correlational selection can be detected across the limited range of variation observed within a single population [63,148,150,151]. Only when populations and species with widely varying blossom sizes are compared is strong phenotypic integration between gland areas and gland–stigma distance revealed [63] (figure 4).

The multi-level approach has a long tradition in the study of allometry, where allometric relationships are studied along individual growth trajectories (ontogenetic allometry), along individual differences at the level of the population (static allometry), and along differences among populations or species (evolutionary allometry; [152]). A working hypothesis for many studies of allometry has been that evolutionary allometries are outcomes of constraints imposed by the lower static, or ontogenetic, levels. This is, for example, manifest in the idea that evolution happens through heterochronic changes along allometric constraints [16,153,154]*.* However, the relationships between these levels are not simple [17,55]. Static allometries are not simple reflections of ontogenetic allometries, and constraining a population to evolve along a static allometry requires that the static–allometric intercept and slope both remains invariant (not evolvable). Static and evolutionary allometries indeed often resemble each other, particularly on the subspecies level [17], but it remains open whether this is caused by constraints or by similarities in selection within and among populations [20].

We suggest that the conceptual framework, theory and hypotheses often studied in relation to allometry can be fruitfully extended to the study of integration more generally. We can recognize ontogenetic, static and evolutionary integration, and it is a goal to understand their interrelationships. Like with allometry, it is clear that this relationship will not be a simple one-to-one mapping.

Several authors have compared integration at multiple levels of organization, for example, ontogenetic versus static integration [144,155], within populations versus among populations [62,63,144,156] and within versus among species [63,157] (see review in Klingenberg [143]). As previously mentioned, the multi-level approach offers the advantage of highlighting risks and weaknesses in the logic of adaptive integration within populations, especially when natural selection has eroded variation and masked patterns of covariation. In such cases, comparisons of population or species might reveal the existence of functional integration that only emerges at higher levels, because differences in population means generate sufficient variation for adaptive covariance to be detected (figure 4; [62,63,143]). By contrast, integration that reflects genetic constraints (e.g. pleiotropy) will usually be detected at both the within- and among-population levels, as will adaptive integration that has been genetically assimilated by evolution of the G-matrix (additive genetic variance matrix).

## 5. Measurement of integration and modularity

There are many experimental and observational methods for studying integration and modularity. Many of these are specific to given experimental systems, and we will not attempt to review them here; instead, we make some general remarks as to how integration and modularity are measured. In this context, we mean measurement in the technical sense of quantification, i.e. how numbers are used to represent an underlying (theoretical) entity (e.g. [158]). Hence, to be able to talk about measurement at all, we need to identify both what is being measured and the actual procedure of measurement. In the study of morphological integration, the former is actually much harder than the latter. There is a whole zoo of quantitative methods and statistics that are used to ‘measure’ integration, but owing to the lack of a well-defined common theoretical framework, it is often hard to say precisely what is being measured by these methods.

For example, there are many different one-dimensional indices of integration in use (table 1; see [164,170] for overviews). The first of these was Olson and Miller's index of morphological integration [5], which consisted of tabulating statistically ‘significant’ correlations between traits and dividing by the number of possible pairwise trait combinations. This measure is unsatisfactory by modern standards, because it relies on statistical significance testing and thus does not capture the strength of correlations, but it also has a more interesting theoretical problem in that it is merely a descriptor of a pattern of correlation with no further connection to theory. Like Cheverud [35], Hallgrímsson *et al*. [8], Mitteroecker *et al.* [48,64] before us, we argued above that the concept of integration is most usefully defined in relation to the underlying developmental and functional mechanisms that created the potential for correlation and not as the correlations themselves. Indeed, Olson & Miller [5] were clearly interested in this connection, and most of their methods were directed toward this end. In their commentary published in a reprinting of the Olson and Miller book, Chernoff & Magwene [6] went as far as defining integration as the correspondence between patterns of covariation and underlying ‘hypotheses’.

**Table 1.** Overview of published indices related to the concept of integration. In the definitions, *N* is the number of traits, *λ* is the eigenvalues of the correlation matrix, *r* is the set of pairwise correlation coefficients, E denotes the expectation (the average) and |*x*| denotes the absolute value of *x*.

index	notes	references
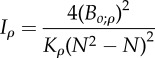	a complex index related to the fraction of correlations above a fixed threshold, and scaled to lie between 0 and 1^a^	Olson & Miller [5]
*I_z_* = tanh(E(|*z*|))	average of Fisher's *z*-transformed correlation back transformed to 0–1 scale^b^	Van Valen [32]
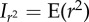	average coefficient of determination, estimated as the mean of the squared pairwise correlations	Van Valen [159]
*I_r_* = E(|*r*|)	average of the absolute pairwise correlations	Cane [160]
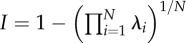	one minus the geometric mean of the correlation-matrix eigenvalues	Cheverud *et al*. [161]
var(*λ*)	variance of the correlation-matrix eigenvalues	Wagner [162], Cheverud *et al*. [163]
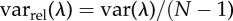	relative variance of the correlation-matrix eigenvalues; the value *N −* 1 is the maximum possible variance of an eigenvalue	Pavlicev *et al*. [164]
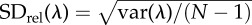	relative standard deviation of the correlation-matrix eigenvalues	Cheverud *et al*. [161], Pavlicev *et al*. [164]
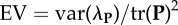	the variance of the variance-matrix eigenvalues ( 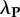 ) scaled by the total variance^c^	Young [165], Willmore *et al*. [166]
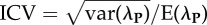	the standard deviation of the variance-matrix eigenvalues scaled by the mean of the eigenvalues	Shirai & Marroig [167]
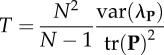	one of Van Valen's [159] measures of ‘tightness’, the closeness of the distribution to the major axis, varying between 0 and 1	Van Valen [159]
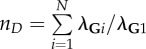	the sum of the genetic variance matrix eigenvalues (*λ*_**G**_) scaled by the leading eigenvalue (*λ*_**G**1_)	Kirkpatrick [168]
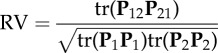	a measure of the total amount of covariation between the two sets of variables over a measure of the total amount of variation in the within the two groups^d^	Klingenberg [98]
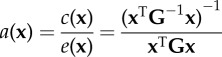	the fraction of independent additive genetic variation (autonomy) for a particular linear combination of the traits (**x**)^e^	Hansen & Houle [169]
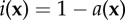	the fraction of non-independent additive genetic variation (integration) in the direction of **x**	Hansen & Houle [169]
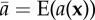	the average autonomy of uniformly distributed random directions^f^	Hansen & Houle [169]
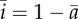	the average integration of uniformly distributed random directions	Hansen & Houle [169]

^a^*B_o;*ρ*_* is number of correlations above, or equal to, the lower statistical significance level (a function of sample size) of a fixed arbitrary threshold correlation given by *ρ*. K_*ρ*_ is the number of non-contained *ρ*-groups, where non-contained means the largest group which can be formed where all elements have pairwise correlations ≥ *ρ*.

^b^tanh is the inverse Fisher transformation (the hyperbolic tangent), *z* is a set of Fisher's *z*-transformed pairwise correlation coefficients.

^c^tr is the trace function. **P** is the phenotypic variance matrix.

^d^**P**_1_, **P**_2_, **P**_12_ and **P**_21_ are the sub matrices of a phenotypic variance matrix. The sub matrices **P**_1_ and **P**_2_ are the variance matrices for the two sets of traits, respectively. The sub matrices **P**_12_ and **P**_21_ are the covariances between the two sets of traits.

^e^*e*(**x**) is the evolvability and c(**x**) the conditional evolvability along a unit length vector (or direction) of the traits **x**, **G** is the additive genetic covariance matrix, ^T^ denotes the transpose, and ^−1^ denotes the inverse. To calculate the autonomy to a specific trait with respect to the rest, we can use a vector **x** with the coefficient 1 for the focal trait and zero for the rest. The different indices from Hansen & Houle [169] are easily computed using the R package ‘evolvability’ (see [156]).

^f^This can be approximated by 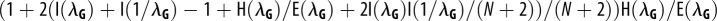, where H(*λ*) ≡ 1/E(1/*λ*) is the harmonic mean, and I(*λ*) ≡ var(*λ*)/E(*λ*)^2^ is the mean-standardized variance. The average autonomy can alternatively be measured as the average of the individual trait autonomies (see [90,114]).

Most indices of integration have the above-described problem of being mere summaries of correlation with no link to formal theory. This is true of Van Valen's [159] mean-squared correlations and Cane's [160] mean absolute correlation, and of various measures based on variances or standard deviations of eigenvalues in the correlation (or variance) matrix [159,161–166]*.* It is important to realize that none of these measures is derived from any model of relationship to either development or evolution. While they are valid descriptors of various aspects of correlation, they are not measures of integration in any sense beyond this. For this reason, it is also impossible to evaluate which one is the best measure of (underlying) integration. The evaluations and comparisons of these indices recently presented by Pavlicev *et al*. [164] and Haber [170] are concerned with their ability to describe or contrast aspects of a given statistical correlation matrix, and not with their ability to capture integration on the developmental, variational, evolutionary or functional levels.

The theoretical literature we discussed above shows that patterns of standing variation and covariation stand in complex relation to organismal structure and evolutionary mechanisms (see also Marquez [97]). For this reason, the correlation-based indices of integration and modularity should not be used to make inferences about either developmental integration or evolutionary potential. Similar considerations apply to many methods aimed at describing patterns of multivariate variation such as principal component analysis or exploratory factor analysis. If such methods are to be informative, they need to be connected to *a priori* hypotheses about the developmental structuring of the variation [11,97,98], or to hypotheses about evolutionary consequences of the variation (evolutionary integration).

Hansen & Houle [169] developed unidimensional measures of evolutionary integration and autonomy derived from measures of evolvability. This approach started with an attempt at quantifying Lewontin's [14] concept of quasi-independence by Hansen *et al*. [22], who did this by defining the *conditional evolvability* of a trait (or set of traits) as their evolvability when other (defined) traits were not allowed to change. In their model, conditional evolvability was shown to equal the conditional additive genetic variance of the trait(s). The conditional variance is the residual variance from a regression on the constraining traits. The conditional evolvability is thus a measurement of evolutionary modularity as above defined. From this, Hansen & Houle [169] proposed to use the autonomy, defined as the ratio between the conditional and the unconditional evolvability, as a relative measure of how much the focal trait is constrained by other traits. The autonomy varies between 0 and 1, with 1 meaning that the traits are completely unconstrained. Their integration measure is simply 1—autonomy. Note that these measures can be defined for any trait or trait vector, and that evolvability, and consequently evolutionary integration, may differ in different directions of morphospace. If an overall measure of (evolutionary) integration is desired, then Hansen & Houle [169] proposed using the average integration over different directions in morphospace.

Many other measures of evolutionary potential can likewise be informative about evolutionary integration and modularity. Schluter's [15] method of comparing species divergence to the direction of maximal evolvability can be used to test whether trait integration acts as a constraint (but see [156,171]). Agrawal & Stinchcombe [172] proposed to test how traits constrain each other by comparing evolvabilties with or without putting trait correlations to zero. Kirkpatrick [168] developed an alternative decomposition showing how trait correlations affect evolvability. Marroig & Cheverud [120] proposed to compute ‘evolutionary flexibility’ defined as the average angle deviance of a predicted selection response from a set of random selection gradients. Although not a direct measure of evolvability, it tells us something about the evolutionary ability to stay on target. We can thus appreciate that there are now several quantitative measures of how patterns of statistical integration influence evolutionary potential.

There are also many methods that are based on comparing patterns of statistical integration to *a priori* hypotheses about developmental structure. Measurement theory tells us that decisions about the attributes to be measured should be based on precise theoretical descriptions of the physical processes that generate these attributes and their relationships. Consequently, these confirmatory methods thus often provide a valid link between integration statistics and developmental or functional hypotheses. Many of the comparisons of *ρ* and *F* statistics in Olson & Miller [5] and tools such as conditional independence [173,174], confirmatory factor analysis [97,131,175], block-correlation methods based on ‘partial least-squares’ [11] and proximity graphs [176] may fall into this category. In the search for valid measurement methods, Mitteroecker & Bookstein [11] first used path models to formalize the relationship between hypothesized developmental systems and the modularity and integration they generate. Using these models, they explored how various statistical methods performed in identifying and measuring integration. Klingenberg [98] showed how the RV coefficient, a measure of statistical modularity in landmark data, could be used to compare different developmental models of *Drosophila* wing modularity with observed patterns of integration. Marquez [97] developed a general statistical framework for combining different hypothetical ‘modules’ of variation. Marquez used this to show how variation in rodent mandibles results from several underlying and partially overlapping developmental modules (as in the ‘palimpsest’ of Hallgrímsson *et al*. [8]). In other cases, however, the link between the measurement and underlying biological questions and theory is weak or absent, as discussed above for integration indices.

Developmental integration can also be studied through patterns of pleiotropy derived from using molecular markers to map quantitative-trait loci (QTLs) or from other genetic data. Several studies test whether patterns of pleiotropy conform to *a priori* hypotheses of developmental or functional integration and modularity [35,102,104,177–182]. In essence, a few QTLs with statistically significant effects are identified, and used to check whether hypothesized integrated traits share QTLs. Of particular interest for the evolution of pleiotropy and integration are studies of differential epistasis [83,84,103,183]. These studies identify relationship QTLs that modify the pleiotropic effects of other QTLs, and thus give evidence for genetic variation in trait covariance. Studies of gene effects suffer from serious methodological problems, however, in particular because of the use of significance testing which leads to strong bias towards genes of large effect [184]*.* In addition, studies of pleiotropy have the measurement/theoretical challenge of sensibly comparing gene effects across different traits, and to develop proper measures of gene effects in the context of modularity and integration. There is, however, a lot of research on developing methods to deal with the problem of not detecting genes of small effects [185–189]. Given that the statistical and theoretical issues can be overcome, studies of gene effects may give us important insights into the genetic architecture of trait integration.

### (a) Defining the characters

Any discussion of integration presupposes an *a priori-*defined set of characters. This gives an element of circularity to its study. The very definition and recognition of a biological character presupposes that it has some level of variational independence [59,190–192]. Characters are integrated modules! This makes it clear that the results of any analysis of integration is highly sensitive to the definition of traits, and also that comparison of and generalization from studies having used different trait definitions are highly problematic [9].

Current biological theory is not sufficiently developed to provide an operational definition of character, and a degree of vagueness is therefore unavoidable. Here, we provide only some general observations. The first concerns the role of size in the analysis. Size itself suffers from a similar vagueness as character (nevertheless see Mosimann [193]), but it is clear that most ratio-scale traits share a strong component of positive covariation along an axis we often call ‘size’. Whether or not such size is included in an analysis of integration will strongly affect the results, and we cannot compare studies that have and have not corrected for size. How to correct for size is another thorny task. Usually, the covariation with size is on a proportional scale, i.e. it is allometric. Correcting for size requires fitting an allometric relationship and the allometric relationships may differ in different systems, making comparison very hard. For example, in their comparison of allometric slopes in different groups, Voje *et al*. [17] encountered the problem that size was often defined from a principal component analysis, and then it was not clear if a difference in allometric slope between populations would be owing to a difference in the size definition or in the allometric slope itself.

In morphometric studies, this problem arises in the choice of landmarks, and conclusions are contingent on the assumption that the landmarks are homologous. The problem of homology has also made it difficult to test the Berg hypothesis with respect to the decoupling of the phenotypic variation between floral and vegetative traits, because this requires a comparison of floral and vegetative traits that are not normally homologous. Just choosing some floral and some vegetative traits and comparing an index of integration may yield a difference, but it is hard to know if this difference is due to the choice of traits or to a real difference in integration. Hansen and co-workers [140,194] tried to circumvent this problem by comparing floral involucral bracts with a function in pollination with partially homologous leaves, but the homology was not perfect, and this will not be possible in most pollination systems. Similarly, Ordano *et al*. [137] pointed out that tests of the hypothesis that flowers with more specialized pollination should be more integrated are inconclusive because they have been based on comparing integration indices over very different systems, including different numbers of non-homologous traits, some of which not being involved in pollen transfer (see also [195,196]).

### (b) Integration and allometry

As noted above, allometry can be viewed as a special case of integration [9,35], and similar to developmental integration, morphological allometry has been considered both a possible constraint on adaptive evolution [17,20,37,38], and itself an adaptation to functional needs [43,197,198]. Thus, studies of allometric covariation are relevant to the development of integration studies.

Morphological allometry can be derived from explicit models of co-regulated growth [17,37,199]*.* This means that its parameters can be given precise biological interpretations on the developmental level. Most pertinently, the narrow-sense allometric slope can be related to the ratio of the co-regulated growth rates of the two traits. This explicitness clarifies the biological meaning of parameters and helps the development of appropriate statistics for their study. For example, the problems related to identifying proper indices for quantifying developmental integration can be solved only by formulating a biological model and identifying integration with parameters in this model. This will not only aid the biological interpretation of the results, but also guide and constrain the statistical methods that can be used. Similarly, the study of the evolution of allometry has been facilitated by the existence of explicit models that can identify the exact targets of selection.

One of the most striking observations about ontogenetic and static allometries is their invariance. Although there are many examples of differences between allometric slopes in related species, the recent review of Voje *et al*. [17] concluded that there is almost no evidence for evolvability of static allometric slopes, nor are there examples of changes that have happened on timescales below millions of years. This is a strong indication that at least some aspects of integration may be severely constrained. On the other hand, changes in allometric relationships may be an important mode of evolution for developmental integration.

Morphological allometry and developmental integration have both been considered to represent possible constraints on adaptive evolution [17,20,37,38]. A major difference, between the two concepts, however, lies in the importance placed on the trajectory of the covariance by allometric studies. While usually only covariances or correlations are used as parameters in studies of phenotypic integration (but see [131]), the slope (i.e. 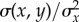) is of major interest in studies of allometry. Although many integration studies err in failing to consider the allometric slope (see discussion in Armbruster *et al.* [131]), many allometry studies err in failing to consider the coefficient of determination (*r*^2^) when assessing relationships between traits [200].

Shallower or steeper allometric slopes do not necessarily indicate weaker or stronger integration, and considering both the allometric slope and the tightness of the relationship (i.e. the integration) may provide additional information about the possible correlational selection generating the covariance among traits or the developmental constraint generated by it. For example, floral canalization has been achieved by some tropical monocot species, despite strong floral–vegetative integration, by the expression of very shallow allometric slopes [131]. Other examples include the difference between static allometric slopes of primary and secondary sexual characters in many insect species. Genitalia of male insects generally display shallow allometric slopes (*β* < 1), a phenomenon explained by the one-size-fits-all hypothesis [201]*.* Armbruster *et al*. [131] reported a similar phenomenon in some tropical flowers. By contrast, horns and other secondary sexual characters display steep allometric slopes [17,202]. However, both relationships are similarly tight (similar *r*^2^; [17]). Together, these observations suggest that assessments of phenotypic integration would be improved by examining the direction of integration or autonomy (*sensu* Hansen & Houle [169]), as well as the strength of the relationship (see discussion in [131]).

## 6. Discussion and conclusions

Integration and modularity are clearly important concepts in evolutionary biology, but as with most heavily used concepts, there is a diversity of opinions as to how best to use and define these terms. Some of this diversity is reflected in the papers that follow in this theme issue. Integration and modularity can refer to the genetic, developmental, evolutionary and functional capacities to covary or not, respectively, but they are also used to describe the statistical properties of traits. These concepts have also been applied at among-population and among-species levels, where definitions can become even broader. For example, the capacity to covary adaptively may only be detected when examining among-population covariation. This complexity has dogged the field as it struggles to find a common language of communication. Another problematic issue is that the evolution of integration is more complicated than usually recognized and is not necessarily just the product of correlational selection. Measurement of integration has been compromised by unclear relevance of indices to theory and uncertainty in what they actually measure owing to complications generated by lack of trait homology and heterogeneity in trait dimensionality and number. Nevertheless, there has been considerable advancement in our empirical knowledge through the development of well-studied systems such as the vertebrate cranium, mandible and limbs, insect wings, and angiosperm flowers.

The papers that follow display a diversity of interpretations of not only the Berg hypothesis, but also key concepts such as integration itself. This plurality reflects the state of the field, so it seems natural to let it stand as exemplary of the present diversity of approaches. Nevertheless, we hope we have begun to build a framework for common terminology and have pointed the way forward for greater synthesis across studies of both plants and animals, and of genes, development, ecology and evolution.

Conner & Lande [130] discuss Berg's contributions to the study of genetics, canalisation and modularity, pointing out that she had far less to say about integration than most people think. Klingenberg [143] reviews biological concepts and analytical methods for characterizing patterns of variation, with emphasis on geometric morphometrics and comparisons across hierarchical levels of organization.

Several of the papers that follow address aspects of floral integration. Vallejo-Marin *et al*. [19] assess the allometric scaling of floral traits in association with the reductions of flower size and reduced heteranthery (anther dimorphisms) in *Solanum* (Solanaceae) clades, finding evidence of repeated parallel transitions of nearly identical nature. A study by Pérez-Barrales *et al*. [203] finds dramatic differences in integration and variance patterns in dimorphic and monomorphic populations of *Narcissus* (Amaryllidaceae); these differences were associated with differences in type and behaviour of the main pollinators. Diggle [138] raises an additional layer of complexity in reminding us that flowers usually vary phenotypically with position in an inflorescence (reflecting both positional and temporal effects), and that we lose insights into floral development and function if flower position is ignored. Diggle also develops the idea that flowers often comprise two or more modules and these may vary in their response to position in the inflorescence; for example, in the *Nicotiana* study, system efficiency (fit-with-pollinator) is usually invariant with position, whereas attraction traits are often variable with position. Ellis *et al*. [204] report correlated variation in fly-mimic traits associated with sexual deception of the main pollinators of *Gorteria diffusa* (Asteraceae) in South Africa. Gómez *et al*. [205] use geometrical morphometrics to quantify integration of flowers of *Erisimum* spp. (Brassicaceae), plants with generalized pollination. Despite the largely generalized pollination, these plants show a trend towards greater integration in populations/species with fewer kinds of pollinators (more specialized pollination). Stock *et al*. [206] explore the covariance patterns of floral and growth traits of *Ipomoea hederacea* (Convolvulaceae) along a latitudinal gradient. They find evidence of polygenic clines in response to shallow environmental gradients. These patterns are consistent with past findings of natural selection on flowering phenology, presumably owing to season-length variation across latitudes. Finally, Bolstad *et al*. [156] assess the role of genetic integration of floral traits (architecture of genetic correlations) in constraining the multivariate trajectory of population divergence. Paradoxically, they find clear evidence of constraint despite estimates of evolvability that are sufficiently large to allow unfettered multivariate evolution.

Transitioning to animals, Conner *et al*. [141] compare patterns of integration across plants and animals, finding evidence that plants (vegetative traits), hemimetabolous insects and vertebrates are similarly integrated, with mean correlations of about 0.5. Holometabolous insects were strikingly more integrated as adults than the other organisms surveyed. Floral and vertebrate-skull traits had similarly low integration, perhaps because both represent multiple modules. This underscores the importance of recognizing modules *a priori*, as emphasized above (see also Murren [7]), rather than simply calculating overall integration across heterogeneous groups. Pitchers *et al*. [207] compare rates of multivariate evolution also across both plants and animals, attempting to assess the role of genetic architecture in limiting the response to selection; they argue that sexually selected traits may have evolved more rapidly than naturally selected traits, and that this reflects differences in evolvability rather than strengths of selection.

Looking at the evolution of the vertebrate skeleton, Goswami *et al*. [142] address questions about the macroevolutionary consequences of patterns of integration and modularity and use a modelling approach to explore the effects of phenotypic integration on rates of evolution and long-term evolutionary trends in disparity (phenotypic diversity). In a focused longitudinal study, Firmat *et al*. [18] examine the evolution of static allometry in rodent toot shape over time, showing that this aspect of integration has remained remarkably invariant over some 600 000 years.
